# Impact of COVID-19 outbreaks and interventions on influenza in China and the United States

**DOI:** 10.1038/s41467-021-23440-1

**Published:** 2021-05-31

**Authors:** Luzhao Feng, Ting Zhang, Qing Wang, Yiran Xie, Zhibin Peng, Jiandong Zheng, Ying Qin, Muli Zhang, Shengjie Lai, Dayan Wang, Zijian Feng, Zhongjie Li, George F. Gao

**Affiliations:** 1 Division of Infectious Diseases, Chinese Center for Disease Control and Prevention, Beijing, China; 2 School of Population Medicine and Public Health, Chinese Academy of Medical Sciences/Peking Union Medical College, Beijing, China; 3 National Institute for Viral Disease Control and Prevention, Chinese Center for Disease Control and Prevention/Chinese National Influenza Center, Beijing, China; 4 WorldPop, School of Geography and Environmental Science, University of Southampton, Southampton, UK; 5 Office of Director, Chinese Center for Disease Control and Prevention, Beijing, China

**Keywords:** SARS-CoV-2, Viral epidemiology, Preventive medicine, Epidemiology

## Abstract

Coronavirus disease 2019 (COVID-19) was detected in China during the 2019–2020 seasonal influenza epidemic. Non-pharmaceutical interventions (NPIs) and behavioral changes to mitigate COVID-19 could have affected transmission dynamics of influenza and other respiratory diseases. By comparing 2019–2020 seasonal influenza activity through March 29, 2020 with the 2011–2019 seasons, we found that COVID-19 outbreaks and related NPIs may have reduced influenza in Southern and Northern China and the United States by 79.2% (lower and upper bounds: 48.8%–87.2%), 79.4% (44.9%–87.4%) and 67.2% (11.5%–80.5%). Decreases in influenza virus infection were also associated with the timing of NPIs. Without COVID-19 NPIs, influenza activity in China and the United States would likely have remained high during the 2019–2020 season. Our findings provide evidence that NPIs can partially mitigate seasonal and, potentially, pandemic influenza.

## Abstract (web summary)

Non-pharmaceutical interventions (NPIs) implemented to interrupt COVID-19 transmission may also impact the spread of other infectious diseases. Here, the authors estimate that influenza activity in China and the United States reduced by up to 80% when NPIs were in place in the 2019–2020 season.

## Introduction

Wuhan Municipal Health Commission reported a cluster of cases of pneumonia on December 31, 2019. A novel coronavirus, later named SARS-CoV-2, was identified on January 7, 2020 as the cause of the cluster^1^. In the United States (the US), the first case was reported on January 20, 2020. World Health Organization named the disease coronavirus disease 2019 (COVID-19) and characterized it as a pandemic in March 2020. COVID-19 is the first pandemic known to be caused by a coronavirus^1,2^; it spread rapidly worldwide, causing great health and socioeconomic damage due to its clinical severity and ease of transmission^3,4^. In the absence of readily available, effective pharmaceutical agents against the emerging virus, countries implemented non-pharmaceutical interventions (NPIs) to contain or slow SARS-CoV-2 transmission. These measures included social distancing and restrictions of personal movement (e.g., canceling mass gatherings, closing public entertainment venues, closing schools, restricting domestic and international travel, and issuing stay-at-home orders); use of individual protection (e.g., wearing masks, practicing good hand hygiene and respiratory etiquette); and social mobilization (e.g., publicity, education, and risk communication)^5,6^. People may have adopted more hygienic lifestyles to avoid COVID-19 infection.

Wuhan city was “locked down” on January 23, 2020 by sharply curtailing in and out traffic. Soon afterward, all provinces in mainland China initiated first-level (highest) emergency responses and adopted stringent NPIs—especially inter-city traffic controls, wearing face masks, and issuing stay-at-home orders^7^. The COVID-19 epidemic was controlled and sustained local SARS-CoV-2 transmission stopped in mainland China by April 2020 with NPIs alone^8^. In the US, following a national emergency declaration issued on March 13, 2020, state governments used NPIs to reduce COVID-19 transmission^9^. By April 1, four US metropolitan areas—Seattle, San Francisco, New York City, and New Orleans—documented significant reductions of new COVID-19 cases after implementing COVID-19 mitigation measures^9^.

Influenza and COVID-19 have similar clinical symptoms and transmission routes^10–12^. Influenza activity is carefully monitored in the US and China through sensitive, laboratory-based surveillance systems^13,14^. In most provinces of China and in the US, rates of influenza laboratory test positivity declined sharply during the winter–spring season of 2019–2020^6,15^. For example, the percent of influenza-positive tests among US respiratory specimens decreased from >20% between January 20, 2020 and March 13, 2020 to 2.3% during the week of March 22, 2020, and remained at historically low inter-seasonal levels after April 5^15^. In contrast, during the same epidemic weeks of the eight influenza seasons during 2011–2019, influenza activity had maintained at a moderate or high level.

NPI-based prevention and control of COVID-19 provided an opportunity to observe the real-world effectiveness of NPIs at mitigating seasonal influenza virus transmission using a comparison study design. Preliminary studies have reported that COVID-19 NPIs may have reduced the spread of influenza viruses^16^, but the evidence was obtained largely from observational modeling studies^17–19^. Comparative studies of the impact of COVID-19 outbreaks and interventions on the intensity of influenza activity are needed to augment current understanding.

In our study, we extracted national sentinel surveillance data on influenza-like illness (ILI) and virological testing results of respiratory specimens across the 31 provinces of mainland China from 2011 to 2020. We also used publicly available data on influenza test results from the US Centers for Disease Control and Prevention (CDC). To quantify the impact of COVID-19 NPIs on influenza, we built time-series models to fit historical influenza data^20^ and compared observed influenza activity in the 2019–2020 season with predicted influenza epidemic levels under a counterfactual scenario of no COVID-19 pandemic and related NPIs. The findings of this study improve our understanding of the efficacy of COVID-19 NPIs at mitigating other respiratory diseases and provide evidence for tailoring control strategies for a future epidemic or pandemic influenza.

## Results

### Influenza activity intensity during the 2019–2020 season in China

Based on influenza virological surveillance test positivity rates from Southern and Northern China during winter–springs of 2011–2019, we classified influenza activity intensity into three levels—high, medium, and low—corresponding to ≥25% laboratory test positive, 20–25% positive, and <20% positive across all epidemic weeks of each monitoring year (see “Methods” for details). Polynomial curves were fit for each influenza activity level by year (Supplementary Table 1). Northern and Southern China had winter–spring epidemic peaks each year from 2011 to 2019. Peak times of the epidemic week in the South were ~2 or more weeks later than in the North (Supplementary Fig. 1).

**Fig. 1:**
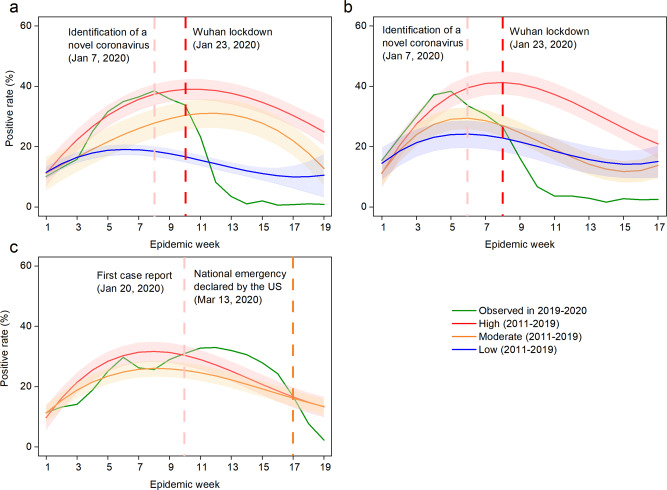
Observed seasonal influenza activity in 2019–2020 and predicted levels using 2011–2019 historical data. **a** Southern China. **b** Northern China. **c** The US. The intensity of influenza activity was divided into three levels in China: high, moderate, and low, corresponding to high (≥25%), moderate (20–25%), and low (<20%) average test positivity rates for all epidemic weeks within a monitoring year from 2011 to 2019, while that of was two levels (high and moderate) in the US under the same classification standard. The fitted curve for each intensity level is presented with lower and upper bounds (shaded color). The pink vertical line indicates when China (**a**, **b**) first identified SARS-CoV-2 and the United States (**c**) first reported COVID-19 cases. The red vertical dashed lines indicate the start of the Wuhan lockdown. The orange vertical line indicates the national emergency declaration by the US. The abscissa represents the epidemic week of winter–spring seasons. The influenza test positivity rates = the number of positive samples of influenza virus test/the number of test samples × 100%.

Before SARS-CoV-2 was confirmed as the cause of the viral pneumonia of unknown etiology cluster in China (January 7, 2020) and NPIs were widely implemented, influenza activity levels in the North and the South were similar to the high epidemic levels observed during the same epidemic weeks in previous years (Fig. 1a, b). Starting January 23, 2020, all provinces initiated their highest level public health emergency response to the COVID-19 outbreak. Influenza activity levels subsequently decreased from high, during epidemic week 10 (Wuhan lockdown) in the South (test positivity rate, 33.8%) and week 8 (Wuhan lockdown) in the North (test positivity rate, 26.5%), to low, during weeks 13–19 in the South (average positive rate: 0.6%) and weeks 11–17 in the North (3.2%) (Fig. 1a, b).

### Influenza activity intensity during the 2019–2020 season in the US

Based on the influenza activity intensity classification criteria above, there were only high and moderate levels found in the US during the 2011–2019 seasons. The US had winter–spring epidemic peaks every year from 2011 to 2019, with stable peak times across years (Supplementary Fig. 1). Before the US declaration of a state of emergency on March 13, 2020, influenza activity in the US was at high or moderate epidemic levels as were observed during the same epidemic weeks in previous years. Influenza activity decreased soon after the declaration (Fig. 1c).

### Impact of COVID-19 and NPIs on influenza in China

**Fig. 2:**
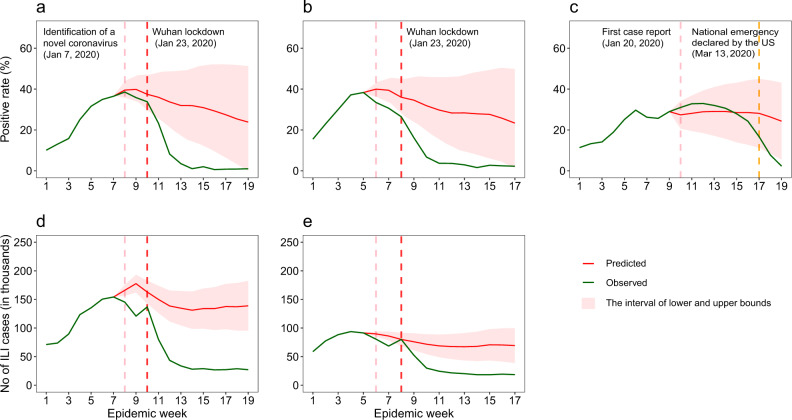
Observed seasonal influenza activity in mainland China and the US in 2019–2020, compared to estimates by ARIMA models under a counterfactual scenario of no COVID-19 and related interventions. **a** Positive rate of influenza tests in Southern China. **b** Positive rate of influenza tests in Northern China. **c** Positive rate of influenza tests in the US. **d** Number (No.) of influenza-like cases reported in Southern China. **e** No. of influenza-like cases reported in Northern China. Lower and upper bounds of estimates are provided. The pink vertical dashed lines indicate when China (**a**, **b** and **d**, **e**) first identified SARS-CoV-2 and the US (**c**) first reported case of COVID-19. The red vertical dashed lines indicate the start of the lockdown in Wuhan, January 23, 2020. The orange vertical dashed line indicates the declaration of a national emergency by the US on March 13, 2020.

**Fig. 3:**
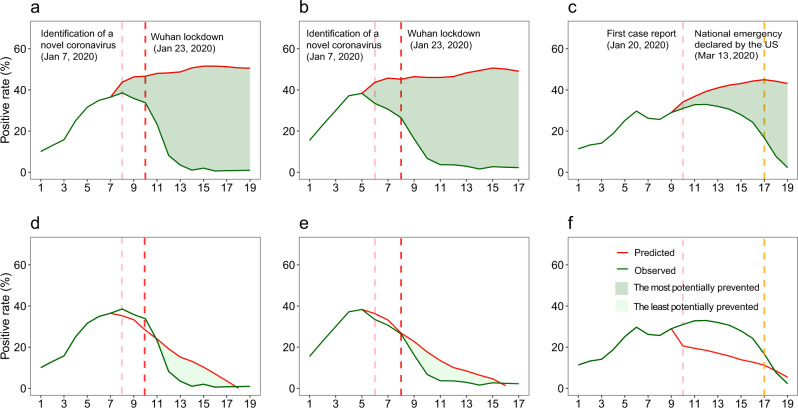
Potential impact of COVID-19 outbreaks and interventions on seasonal influenza intensities in mainland China and the US, 2019–2020. **a**–**c** Comparisons of observed influenza activities with the upper bounds predicted with 2011–2019 expectations under a counterfactual scenario of no COVID-19 outbreaks and related interventions in Southern China (**a**), Northern China (**b**), and the US (**c**). **d**–**f** Comparisons of observed influenza activities with the upper bounds of estimates under the counterfactual scenario in Southern China (**d**), Northern China (**e**), and the US (**f**). The pink vertical dashed lines indicate when China identified SARS-CoV-2 and the US first reported cases of COVID-19. The red vertical dashed lines indicate the start of the lockdown in Wuhan, January 23, 2020. The orange vertical dashed lines indicate the declaration of a national emergency by the US on March 13, 2020. Potentially prevented in influenza activity = (area under the predicted epidemic curve without COVID-19 outbreaks and NPIs − area under the observed epidemic curve)/area under the predicted epidemic curve without COVID-19 outbreaks and NPIs × 100%.

**Fig. 4:**
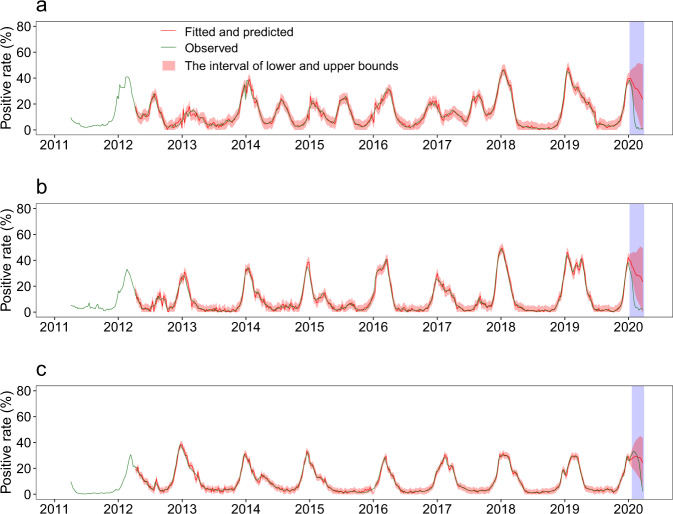
Observed, fitted, and predicted influenza test positivity rate from 2011 to 2020. **a** Southern China. **b** Northern China. **c** The US. The blue shaded parts indicate the estimates under normal seasonal influenza activities and shows 95% confidence intervals of estimates.

We built autoregressive integrated moving average (ARIMA) models to fit influenza activity from 2011 to 2019 and predict influenza epidemic levels during 2019–2020 under a counterfactual scenario in which the COVID-19 pandemic did not occur and therefore strict NPIs were not used (Supplementary Figs. 2–9 and Table 2). In both Southern and Northern China, observed influenza activity levels in the 2019–2020 season were significantly lower than predicted (Fig. 2). In terms of test positivity rates, compared with predicted rates under the counterfactual scenario, influenza activity in Southern China declined by 8.1% (lower and upper bounds: 0–21.3%) during epidemic weeks 8 and 9—the time from identification of the novel coronavirus to the week before Wuhan lockdown—but activity markedly decreased by 79.2% (48.8–87.2%) in weeks 10–19—the time of widespread NPI implementation (Figs. 3 and 4 and Table 1). A similar pattern was found in Northern China, with a slight decrease of influenza activity of 21.7% (6.3–32.8%) before massive NPIs, followed by a marked decline by 79.4% (44.9–87.4%) during widespread NPI implementation. ARIMA analyses showed that 59.7% (49.1–66.6%) and 50.0% (31.6–60.6%) of ILI cases were prevented in Southern and Northern China, respectively (Fig. 2d, e).

**Table 1:** Potential impact of COVID-19 outbreaks and non-pharmaceutical interventions on seasonal influenza activities.

Time Period	Southern China	Northern China	The United States
Period I^a^	8.1 (0–21.3)	21.7 (6.3–32.8)	6.0 (0–23.9)
Period II^b^	79.2 (48.8–87.2)	79.4 (44.9–87.4)	67.2 (11.5–80.5)
Overall	63.5 (30.4–76.0)	66.4 (29.6–78.0)	18.0 (1.5–40.8)

Note: The numbers presented here are the decreases in the positive rate of influenza tests (%), to reflect the impact of COVID-19 outbreaks and interventions on influenza activities. The numbers within brackets represent the lower and upper bounds of estimates.

^a^Period I: for China, it was the time period from the week when the novel coronavirus was first identified to the week before the Wuhan lockdown on January 23, 2020; for the United States (US), it was the time period from the week when the first COVID-19 case in the US was reported on January 20 to the week before the national emergency declared on March 13, 2020.

^b^Period II: for China, it was the time period from the week when Wuhan was “locked down” on January 23 to the week ending on March 29, 2020; for the US, it was the time period from the week when the national emergency was declared on March 13 to the week ending on March 29, 2020.

### Impact of NPIs and timing on influenza in the US

We used ARIMA models to analyze variation in influenza activity in the US during the same epidemic weeks that we used in our Southern China analysis. Prior to March 13, 2020—the US declaration of a state of emergency (epidemic week 17), there were no significant changes in the intensity of influenza activity in the 2019–2020 winter–spring season when compared to the seasonal levels of influenza determined from the US historical data (Fig. 1c and 4c). Influenza test positivity during the 3 weeks following epidemic week 17 decreased by 67.2% (lower and upper bounds: 11.5–80.5%) from predicted levels under the counterfactual scenario, and declined by only 6.0% (0–23.9%) during epidemic weeks 10–16 (Figs. 2c, 3c, f and Table 1).

### Model validation

To evaluate the accuracy and reliability of our model predictions, we used the data of test positivity rates from 2011 through the 2017–2018 season to predict seasonal influenza activity in the 2018–2019 season—the actual situation, and prior to COVID-19. Based on variation between observed and predicted values, we found that ARIMA models had good predictive performances for test positivity rates in Southern China (mean absolute percentage error: 19.5%), Northern China (mean absolute percentage error: 37.7%), and the US (mean absolute percentage error: 16.9%) (Supplementary Fig. 2).

## Discussion

Our study found that decreases in influenza infections were associated with the implementation and timing of COVID-19-related NPIs in China and the US. The model accurately and reliably predicted the 2011–2018 season, lending confidence to our findings. Influenza activity decreased by 67.2 to 79.4% compared with pre-COVID-19 influenza seasons. Had NPIs against COVID-19 not been implemented, influenza activity in China would likely have remained high during the entire 2019–2020 season, as shown in Fig. 2. US virologic surveillance^15^ and similar surveillance in the Northern Hemisphere^19^ showed a consistent, seasonal pattern of influenza before COVID-19. In the absence of readily available and effective pharmaceutical interventions, the adoption of NPIs may be a feasible and effective method to mitigate transmission of emerging respiratory infections, including pandemic influenza^21^.

The rapid decrease and sustained low level of influenza in China during the COVID-19 outbreak could largely be attributed to widespread implementation of NPIs during and after the Wuhan lockdown that started January 23, 2020 (epidemic week 10 in Southern China and epidemic week 8 in Northern China). Influenza activity decreased in a similar fashion in the US after epidemic week 17, and the decrease may be related to the adoption of NPIs after the national emergency declaration on March 13, 2020. It is also plausible that people began to use self-protective behaviors and improved personal hygiene to avoid COVID-19, and that these habits may have contributed to the observed reduction of influenza activities—especially before government-driven NPIs. For example, the gradual decline of influenza activities during weeks 2 to 3 in 2020, before the Wuhan lockdown, might be related to changes in personal behavior—wearing masks, for example—based on government guidelines and recommendations^22^. In addition, COVID-19 first occurred in Southern China, and COVID-19 NPIs were implemented earliest there^22^. The peak of season influenza epidemic usually arrives earlier in Southern China than in Northern China (Supplementary Fig. 1), providing another plausible reason for the coincidence of the decline in influenza with the rise in NPIs in China.

Other COVID-19 research can help illuminate the relation between NPIs and virus transmission. Several interventions have been shown to reduce the spread of COVID-19 by substantially mitigating the spread of the coronavirus^23–26^. Human mobility may have played a critical role in the transmission dynamics of COVID-19, while strict restrictions on international travel have substantially reduced the spread of the coronavirus^21^. Physical distancing, such as canceling mass gatherings, and closing schools, as implemented in China during the outbreak, appeared to have a major impact on containment of the first wave of COVID-19^27^. Proactive school closures reduced the peak incidence of COVID-19 by 40–60% and slowed the pace of the epidemic^27^. Combinations of interventions, implemented early, achieved the strongest and most rapid effect^8^, demonstrating a synergistic effect among stringent NPIs to lower the effective reproduction number of the coronavirus^28^.

Studies in Asia, the US, and Europe have shown that influenza activity declined in 2020 after the first set of measures to fight COVID-19 were implemented^19,29^. The number of ILI cases in China decreased with implementation of NPIs and further declined with increased intensity of intervention measures. Reduction of symptom-based ILI could also be due to decreases in the clinic and hospital visits during the COVID-19 outbreak. Compared with China, the somewhat smaller apparent impact of COVID-19 NPIs on influenza seen in the US data may be due to differences in implementation of COVID-19 interventions between the two countries; to the later arrival of COVID-19 in the US so that that a smaller proportion of the seasonal influenza epidemic (weeks 17–19) overlapped with COVID-19, thus weakening the observed NPI–influenza relationship during the 2019–2020 influenza season; to the inclusion of data from public health laboratories, which are often used for influenza confirmation and may artificially increase the percent positive for influenza; or that a larger proportion of the US population receives seasonal influenza vaccine than the China population, thus lessening influenza more in the US than China and therefore lowering the potential impact of NPIs. Further study is indicated^9^.

There are several limitations of our study. First, virological surveillance was affected by factors such as specimen collection rates and case selection biases, and symptom-based surveillance of ILI could have been affected by circulating virus strains, clinical diagnosis, and healthcare-seeking behaviors, unpredictably changing the observed test positivity rate. Second, our study was limited to the 2019–2020 influenza season through March 29, 2020. Longer inter-seasonal virological and ILI influenza data during COVID-19 outbreaks could be used to further explore the COVID-19 NPI–influenza relationship. Third, the genetic diversity of influenza viruses and their antigenic characteristics were not considered in this study. For example, the influenza virus that circulated in the Northern Hemisphere from October 2018 to May 2019 was dominated by influenza A(H1N1), but the proportion of A(H3N2) viruses increased over time^30^. Fourth, although ARIMA, as used to forecast infectious disease, is a mature and applicable technology, infectious diseases transmission factors such as the type of influenza strain, genetic factors, control measures, and personal activities and behaviors cannot be separately distinguished. ARIMA may not be optimal for a long-term prediction, limiting our confidence beyond short-term predictions.

Evidence from our study improves the understanding of the impact of COVID-19 and COVID-19-related NPIs on the transmission of the influenza virus. It will be critically important to assess the independent and synergistic impact of specific NPI measures on influenza activity, especially since some NPIs have great socioeconomic costs and may not be acceptable to the public or government for mitigating seasonal or pandemic influenza.

## Methods

### Case and epidemic period definitions

Individuals considered to have ILI had a temperature ≥38.0 °C and either cough or sore throat. The average weekly test positive rate was calculated as the number of samples positive for influenza divided by the total number of samples tested during the week. Our study defined influenza epidemic and nonepidemic periods using the same thresholds as previous studies^31–33^. The start of an influenza epidemic period was defined as the first week during which the average weekly test positive rate was >10% and remained >10% for at least two consecutive weeks. The end of an influenza epidemic period was defined as the last week during which the positive rate was <10% and remained >10% for at least two consecutive weeks. The duration of an epidemic season was defined as the number of weeks between the start and the end of an influenza epidemic period. In the 2019–2020 influenza season, the epidemic period started on the 47th week in Southern China and 49th week in Northern China.

### Data and sample sources

We obtained virological and ILI surveillance data in China from the National Influenza Surveillance Network in 2011–2020. The National Influenza Surveillance Network in mainland China, led by China CDC, has 554 sentinel hospitals and 407 network laboratories. Influenza activity levels and trends are monitored using ILI data from surveillance units collected at sentinel hospitals. The Influenza Network Laboratory monitors the etiology of influenza virus from respiratory specimens, which not only include ILI patients from influenza surveillance sentinel hospitals but also include samples collected during influenza outbreaks. In China, weekly virological and ILI data, based on influenza sentinel surveillance, are systematically collected as a proxy of influenza activity. Every 12-month interval, from the 14th week in 1 year to the 13th week of the following year constitutes a surveillance year^14,34^.

We also obtained publicly available influenza virological data in 2011–2020 released by US CDC^13^. In the US, the Influenza Surveillance Network, led by US CDC, contains ~100 public health laboratories and over 300 clinical laboratories^13^. Clinical laboratories primarily test respiratory specimens for diagnostic purposes and provide information on the timing and intensity of influenza activity. Public health laboratories test specimens from clinical laboratories for surveillance purposes to understand influenza virological information, such as the virus types, subtypes, and lineages that are circulating. The total number of respiratory specimens tested for influenza and the number positive for influenza viruses are reported from public health and clinical laboratories to CDC each week^35^.

The positive test rate of influenza in China was calculated from a total of 3,728,252 samples; the positive test rate for the US was determined from a total of 8,349,337 samples >9 years.

### Influenza activity level definitions

Based on influenza test positivity rates, we categorized the average positivity across all epidemic weeks of a monitoring year into high (positive rate ≥25%), moderate (20–25%), and low (<20%) levels. We developed epidemic curves for each level in the winter**–**spring seasons. Because influenza epidemiologic characteristics differ between Southern and Northern China^10,32^, we analyzed data by region. We fit polynomial curves for each influenza epidemic level prior to COVID-19 in 2011–2019 for Southern and Northern China (Supplementary curve fitting, and Supplementary Fig. 1 and Supplementary Table 1).

We compared fitted activity levels in 2011–2019 with observed activity in the winter–spring epidemic weeks in 2019–2020 before the COVID-19 outbreaks and the implementation of NPIs. We then determined the predicted influenza activity by intensity level under a counterfactual scenario of no COVID-19 and NPIs. We investigated influenza infections based on key dates for NPIs in China and the US: January 23, 2020—Wuhan’s lockdown—as the start of strict and combined NPIs in China; March 13, 2020—when a state of national emergency was declared by the US—as the start of NPIs in the US.

### Time-series models

The ARIMA (*p*, *d*, *q*) model is a time-series forecasting method that extends the autoregressive (AR), moving average (MA), and ARMA models^20,36^. It aims to solve two problems: one is to decompose randomness, stationarity, and seasonality of time series; the other is to select an appropriate model for forecasting based on analysis of time series. ARIMA has been widely used to forecast short-term effects and trends of acute infectious diseases^36^. The parameters *p*, *d*, and *q* represent the order of AR, the degree of differencing of the original time series, and the order of the MA, respectively. Due to the seasonality of influenza, we utilized a seasonal ARIMA (SARIMA [*p*, *d*, *q*][P, D, Q]s) model. In SARIMA, P, D, Q, and s refer to seasonal autoregression, seasonal integration, seasonal moving average, and seasonal period length.

- *Sequence stationarity*: time sequences (test positivity rates in Southern and Northern China and the US, and the number of ILI cases in Southern and Northern China) were nonstationary (Supplementary Fig. 3). Sequence stationarity was tested with the augmented Dickey–Fuller test. If lags were outside the confidence intervals after the first three lags, the time sequence was considered nonstationary. After 1-time difference and 1-time seasonal difference, the data sequence is stable with the mean value fluctuating around the indication. (Supplementary Fig. 4).
- *Sequence randomness*: according to the Box–Ljung statistical test results (*p* < 0.05), the hypotheses of independence of the 5-time sequences were all rejected.
- *Identification*: depending on the seasonal decomposition, SAF (seasonal adjustment factors), referring to factors of the seasonal cycle that affect the sequence (Supplementary Fig. 5). ERR (error sequence), referring to the sequence remaining after removing seasonal factors, long-term trends, and cyclic changes from the time series, was ~0 (within 5) and distributed as white noise (Supplementary Fig. 6).
  Through observing the autocorrelation function (ACF) (Supplementary Fig. 7) and partial ACF (PACF) (Supplementary Fig. 8) to recognize and analyze the characteristics of the sequence, we first listed the parameters that met the characteristic of ACF and PACF, and then optimized the parameters in accordance with Akaike information criterion and *R*^2^. In addition, AR model describes the relationship between the current value and the historical value. Since the positive rate of influenza is related to the characteristics of the virus in the epidemic season and the serial interval of influenza is 2–3 days^7^, AR was selected as order 1. Generally, as the duration of influenza immunity antibody is <1 year^37^, it may affect the intensity of influenza activity in the next year. We chose 0–1 for seasonal autocorrelation, but we only presented the top three candidate models in Supplementary Table 2.
- *Estimation and validation*: rationality of the model was assessed by examination of standard model fitting residuals. If fitting residuals of a model for sequences of this study were normally distributed with zero as the mean, and the lag order residuals of ACF and PACF were within confidence intervals (Supplementary Fig. 9), the model was regarded as qualified. To further validate the predictive ability of the model, we also used the influenza data from 2011 to 2018 as a training set to build models and predict the influenza activities for the 2018–2019 season. Results were assessed by comparing the test dataset of observed values in 2018–2019 and the mean absolute percentage errors (Supplementary Fig. 2).
- *Application forecasting*: we used these models with data from 2011 to 2019 to estimate the weekly influenza positivity rate for the winter–spring season in 2019–2020 under a counterfactual scenario with no COVID-19 outbreaks and no COVID-19 NPIs. For China, forecasting started from the week of January 7, 2020 when the SARS-CoV-2 was first identified, corresponding to epidemic week 8 in Southern China and epidemic week 6 in Northern China. For the US, the first week for estimating was the week beginning on January 20, 2020, corresponding to epidemic week 10 in the US. The overall impact of COVID-19 outbreaks and interventions on influenza was defined as the difference in the area between the observed epidemic curve and the model-predicted curve. The upper/lower bounds of estimates were defined as the difference between the observed curve and the model-predicted upper/lower bound curve of confidence intervals. We also assessed the effectiveness of COVID-19 outbreaks and interventions by time period (Table 1), according to the timings of the first identification of SARS-CoV-2 and the implementation of strict NPIs in China, and the dates of the first COVID-19 confirmed case reported and the national emergency declared in the US. Descriptive statistics and time-series analyses were conducted using SAS JMP Pro 14 and SPSS 22.0. The 2019–2020 curve area difference for assessing the NPIs effectiveness used Graphpad prism 8.0. R version 3.6.1 (R Foundation and Origin 2019 for Statistical Computing, Vienna, Austria) was used to plot figures.

### Reporting summary

Further information on research design is available in the Nature Research Reporting Summary linked to this article.

## Supplementary information

Supplementary Information

Reporting Summary

## Data Availability

The influenza virological surveillance data in the US used in this study are publicly available at: https://www.cdc.gov/flu/weekly/fluactivitysurv.htm. All other data associated with this work are available at https://zenodo.org/record/4573183#.YD5JWGgzZdg. All relevant data are available from the authors.

## Code availability

R code for plotting figures in this study is available at https://zenodo.org/record/4573183#.YD5JWGgzZdg

## References

[1] World Health Organization. *Timeline: WHO’s COVID-19 Response* (accessed 20 December 2020); https://www.who.int/emergencies/diseases/novel-coronavirus-2019/interactive-timeline.

[2] The Novel Coronavirus Pneumonia Emergency Response Epidemiology Team. (2020). The epidemiological characteristics of an outbreak of 2019 Novel Coronavirus Diseases (COVID-19)—China, 2020. China CDC Wkly.

[3] Tan W (2020). A novel coronavirus genome identified in a cluster of pneumonia cases—Wuhan, China 2019−2020. China CDC Wkly.

[4] Yang J (2020). Disease burden and clinical severity of the first pandemic wave of COVID-19 in Wuhan, China. Nat. Commun..

[5] Zhu N (2020). A novel coronavirus from patients with pneumonia in China, 2019. N. Engl. J. Med..

[6] Sun J, Shi Z, Xu H (2020). Non-pharmaceutical interventions used for COVID-19 had a major impact on reducing influenza in China in 2020. J. Travel Med..

[7] Li Z (2020). Active case finding with case management: the key to tackling the COVID-19 pandemic. Lancet.

[8] Lai S (2020). Effect of non-pharmaceutical interventions to contain COVID-19 in China. Nature.

[9] Lasry A (2020). Timing of community mitigation and changes in reported COVID-19 and community mobility—four U.S. Metropolitan areas, February 26-April 1, 2020. Morb. Mortal. Wkly Rep..

[10] Shaman J, Kohn M (2009). Absolute humidity modulates influenza survival, transmission, and seasonality. Proc. Natl Acad. Sci. USA.

[11] Li Y (2019). Global patterns in monthly activity of influenza virus, respiratory syncytial virus, parainfluenza virus, and metapneumovirus: a systematic analysis. Lancet Glob. Health.

[12] Wu F (2020). A new coronavirus associated with human respiratory disease in China. Nature.

[13] US Centers for Disease Control and Prevention. *Past Weekly Surveillance Reports* (accessed 30 October 2020); https://www.cdc.gov/flu/weekly/pastreports.htm.

[14] National Health Commission of China. *Notice on Printing and Distributing the National Influenza Surveillance Program (2017 Edition). 39* (accessed 6 July 2020); http://www.nhc.gov.cn/jkj/s3577/201704/ed1498d9e64144738cc7f8db61a39506.shtml.

[15] Olsen SJ (2020). Decreased influenza activity during the COVID-19 pandemic-United States, Australia, Chile, and South Africa, 2020. Am. J. Transplant..

[16] Ryu S (2020). Nonpharmaceutical measures for pandemic influenza in nonhealthcare settings-international travel-related measures. Emerg. Infect. Dis..

[17] Fong MW (2020). Nonpharmaceutical measures for pandemic influenza in nonhealthcare settings-social distancing measures. Emerg. Infect. Dis..

[18] Sakamoto H, Ishikane M, Ueda P (2020). Seasonal influenza activity during the SARS-CoV-2 outbreak in Japan. JAMA.

[19] Fricke LM, Glöckner S, Dreier M, Lange B (2021). Impact of non-pharmaceutical interventions targeted at COVID-19 pandemic on influenza burden - a systematic review. J. Infect..

[20] Benvenuto D, Giovanetti M, Vassallo L, Angeletti S, Ciccozzi M (2020). Application of the ARIMA model on the COVID-2019 epidemic dataset. Data Brief..

[21] Lai, S. et al. Assessing the Effect of Global Travel and Contact Restrictions on Mitigating the COVID-19 Pandemic. *Engineering* 10.1016/j.eng.2021.03.017 (2021) 10.1016/j.eng.2021.03.017 PMC8099556 33972889

[22] The State Council Information Office of the People’s Republic of China. *Full Text: Fighting COVID-19: China in Action* (accessed 7 June 2020). http://www.scio.gov.cn/zfbps/32832/Document/1681809/1681809.htm.

[23] Gibbs H (2020). Changing travel patterns in China during the early stages of the COVID-19 pandemic. Nat. Commun..

[24] Yang J (2020). Uncovering two phases of early intercontinental COVID-19 transmission dynamics. J. Travel Med..

[25] Brauner JM (2021). Inferring the effectiveness of government interventions against COVID-19. Science.

[26] Huang, B. et al. Integrated vaccination and physical distancing interventions to prevent future COVID-19 waves in Chinese cities. *Nat. Hum. Behav*. 10.1038/s41562-021-01063-2 (2021). 10.1038/s41562-021-01063-2 33603201

[27] Zhang J (2020). Changes in contact patterns shape the dynamics of the COVID-19 outbreak in China. Science.

[28] Tian L (2021). Harnessing peak transmission around symptom onset for non-pharmaceutical intervention and containment of the COVID-19 pandemic. Nat. Commun..

[29] Flaxman S (2020). Estimating the effects of non-pharmaceutical interventions on COVID-19 in Europe. Nature.

[30] Chinese National Influenza Center. *Weekly Influenza Surveillance Report from October 2018 to May 2019* (accessed 30 October 2020); http://www.chinaivdc.cn/cnic/zyzx/lgzb/.

[31] Lei, H. et al. Different transmission dynamics of COVID-19 and influenza suggest the relative efficiency of isolation/quarantine and social distancing against COVID-19 in China. *Clin. Infect. Dis.*10.1093/cid/ciaa1584 (2020). 10.1093/cid/ciaa1584 PMC7665384 33080000

[32] Liu XX (2017). Seasonal pattern of influenza activity in a subtropical city, China, 2010-2015. Sci. Rep..

[33] Azziz Baumgartner E (2012). Seasonality, timing, and climate drivers of influenza activity worldwide. J. Infect. Dis..

[34] Wang C, Horby PW, Hayden FG, Gao GF (2020). A novel coronavirus outbreak of global health concern. Lancet.

[35] US Centers for Disease Control and Prevention. *Influenza Surveillance System: Purpose and Methods* (accessed 30 October 2020); https://www.cdc.gov/flu/weekly/overview.htm.

[36] Weng RX (2020). Time series analysis and forecasting of chlamydia trachomatis incidence using surveillance data from 2008 to 2019 in Shenzhen, China. Epidemiol. Infect..

[37] Davis CW (2020). Influenza vaccine-induced human bone marrow plasma cells decline within a year after vaccination. Science.

