# Supplementary material for: Impact of COVID-19 outbreaks and interventions on influenza in China and the United States
Source: Nat Commun. 2021 May 31;12:3249. doi: 10.1038/s41467-021-23440-1 (PMC8167168; doi:10.1038/s41467-021-23440-1)
Supplement: Supplementary file 1 — Supplementary Information [file 41467_2021_23440_MOESM1_ESM.pdf]

Supplementary Information

Impact of COVID-19 outbreaks and interventions on influenza in China and the United States

Luzhao Feng, PhD, Ting Zhang, PhD, Qing Wang, BSc, Yiran Xie, BSc, Zhibin Peng, MSc, Jiandong Zheng, PhD, Ying Qin, PhD, Muli Zhang, MSc, Shengjie Lai, PhD, Dayan Wang, PhD, Zijian Feng, PhD, Zhongjie Li, PhD, and George F. Gao, PhD

Supplementary data:

Supplementary note 1: Data collation and analysis..2

Supplementary note 2: Polynomial curves fitting... 3

Supplementary note 3: ARIMA model for influenza test positive rate prediction4

Supplementary note 4: Evaluating the impact of COVID-19 outbreaks and NPIs on influenza... 7

Supplementary Figures... 8

Supplementary Tables... 16

Supplementary note 1: Data collation and analysis

The influenza laboratory test positive rate for each epidemic week was used to define the intensity of influenza activity. Influenza activity intensity levels were determined by the epidemic weeks in the winter-spring seasons in China and the United States (US). Epidemiological characteristics of influenza vary across China due to geographical differences between the North and the South[1]. Data integrity and availability also varied by province. We conducted analyses for the South part (Jiangsu, Anhui, Zhejiang, Shanghai, Hubei, Hunan, Jiangxi, Fujian, Tibet, Yunnan, Guizhou, Sichuan, Chongqing, Guangxi, Guangdong, Hainan) and the North part (Shandong, Henan, Shanxi, Shaanxi, Gansu, Qinghai, Xinjiang, Hebei, Tianjin, Beijing, Inner Mongolia, Liaoning, Jilin, Heilongjiang, Ningxia) of mainland China.

Intensity of winter-spring influenza activity in 2011-2019 was divided into three levels: high, moderate, and low, corresponding to high (positive rate ⩾25%), moderate (20%–25%), and low (<20%) positive rates of influenza tests, respectively (see the criteria in Methods). Three typical pre-COVID-19 epidemic levels in the North and South of China and two levels in the US were selected for comparison of the observed activity in winter-spring epidemic weeks of 2019-2020, the season that overlapped COVID-19.

Supplementary note 2: Polynomial curves fitting

Curve fitting is the process of constructing a curve or mathematical function that has the best fit to a series of data points, subject to constraints. The order of the equation is a third degree polynomial:

$$y=ax^3+bx^2+cx+d$$

First, the influenza test positive rate in 2011-2019 winter-spring epidemic week was calculated to determine the season corresponding to high, moderate, and low activity intensities described above.

Second, curve fitting functions in SPSS22.0 and SAS JMP Pro 14 were used to fit influenza activity levels at each intensity for southern China, northern China, and the US (Supplementary Fig. 1 and Table S1).

Supplementary note 3: ARIMA model for influenza test positive rate prediction

The ARIMA model was used to predict typical winter-spring epidemic weekly curves that would have occurred in the North and South in 2019-2020 in the counterfactual scenario of no COVID-19 and related NPIs. The observed (actual) curve for the 2019-2020 season was compared with the counterfactual curve to evaluate NPI effectiveness. The model we obtained has the form:

$$\Phi_P\ (B^s)\,\varphi_p\,(B)\,\nabla_s^D \nabla_{Z_t}^d\ = \theta_q\,(B)\,\Theta_Q\,(B^s)\,a_t$$

where

$\Phi_P\ (B^s)=(1-\Phi_1 B^s-\ldots-\Phi_P B^{sP})$ is the seasonal AR operator of order P;

$\varphi_p = (1 - \varphi_1 B - \ldots -\varphi_p B^P)$ is the regular AR operator of order p;

$\nabla_s^D = (1 - B^s)^D$ represents the seasonal differences and represents the seasonal differences and $\nabla^{\mathrm{d}}=(1-\mathrm{B})^{\mathrm{d}}$the regular differences;

$\Theta_Q\,(B^s)= (1-\Theta_1 B^s-\ldots-\Theta_Q B^{sQ})$ is the seasonal moving average operator of order Q;

$\theta_q\,(B)= (1-\theta_1 B-\ldots-\theta_q B^q)$ is the regular moving average operator of order q;

a_t is a white noise process.

Basic principles

The data sequence formed by the predicted object over time is regarded as a non-random sequence. A time series is a group of time-dependent variables[3]. The dependence or auto-correlation of this group of variables represents the continuity of the development of the predicted object. Once this autocorrelation is described by the corresponding mathematical model, the future value can be predicted from the past and present values of the time series[4].

Formula

The general expression of the ARIMA model is ARIMA (p, d, q) (P, D, Q) s,

where p and q are the auto-regressive (AR) and moving average (MA) orders, and P and Q seasonal auto-regression and moving average order, d, D are the difference order and seasonal difference order, s is the seasonal period[5,6].

Modeling steps

① Sequence stationarity and randomness. ARIMA should be built on a stationary sequence. The time series auto-correlation function was used to judge the stationarity[7] of the data in the epidemic week from 2011 to 2019 (Supplementary Fig. 3) and determine the difference d (Fig. S4), D and the seasonal periods[8]. If the auto-correlation function of the time series was greater than 3 and falls within the confidence interval and gradually approaches zero, then the time series is stationary. There is no stationarity if it falls outside the confidence interval, in which case we need to smooth the data to be a stationary by making a difference[9]. A non-stationary series with seasonality into a stationary one by using the transformation:

$$W_t = \nabla_s^D \nabla_{Z_t}^d$$

where D is the number of seasonal differences and d is the number of regular differences.

With a 1-time non-seasonal difference and a 1-time seasonal difference, the sequence non-stationarity was eliminated with white noise. Ljung-Box test determined the Sequence to be non-random (p<0.05).

② Model identification. We determined the model parameters, referring to the sequence scatter plot, auto-correlation function (ACF) plot, and partial auto-correlation function (PACF) plot (Figs. S7–S8). We used Akaike information criterion (AIC) and decision coefficient R^2 from all candidate models to identify the best p and q values, and then determined the parameters P and Q step-by-step[10].

③ Model estimation and validation. We performed a Q test on the residuals to check the autocorrelation of the residuals (Supplementary Fig. 9). If the residuals have no autocorrelation, the model fits well[11].

④ Model fitting and prediction. Based on the data from 2011 to 2019, we observed, fitted and predicted changes in the positive rate of influenza testing and the number of influenza-like cases under the counterfactual scenario of no COVID-19 and related non-pharmaceutical interventions. The goodness-of-fit to the ARIMA model was evaluated by determining the coefficient R^2 (0–1) and AIC. The larger the R^2, the smaller the AIC, the better the fit (Table S2).

Supplementary note 4: Evaluating the impact of COVID-19 outbreaks and NPIs on influenza

The percent decrease in influenza activity = (estimated area under the epidemic curve when no NPIs were taken - area under the observed epidemic curve with NPIs) / Estimated area under the number curve without any NPIs * 100%. *Graphpad prism8.0* was used to calculate the area under the curve to evaluate the impact of the measures. The uncertainty of estimates is defined as:

Upper bound = (the area formed by the upper limit of the estimates by ARIMA model and the *X* axis - the area formed by the observed value and the *X* axis) / the area formed by the upper limit of the estimated value of the model and the *X* axis.

Lower bound = (the area formed by the lower limit of the estimates by ARIMA model and the *X* axis - the area formed by the observed value and the *X* axis) / the area formed by the lower limit of the estimated value of the model and the *X* axis.

Supplementary Figures

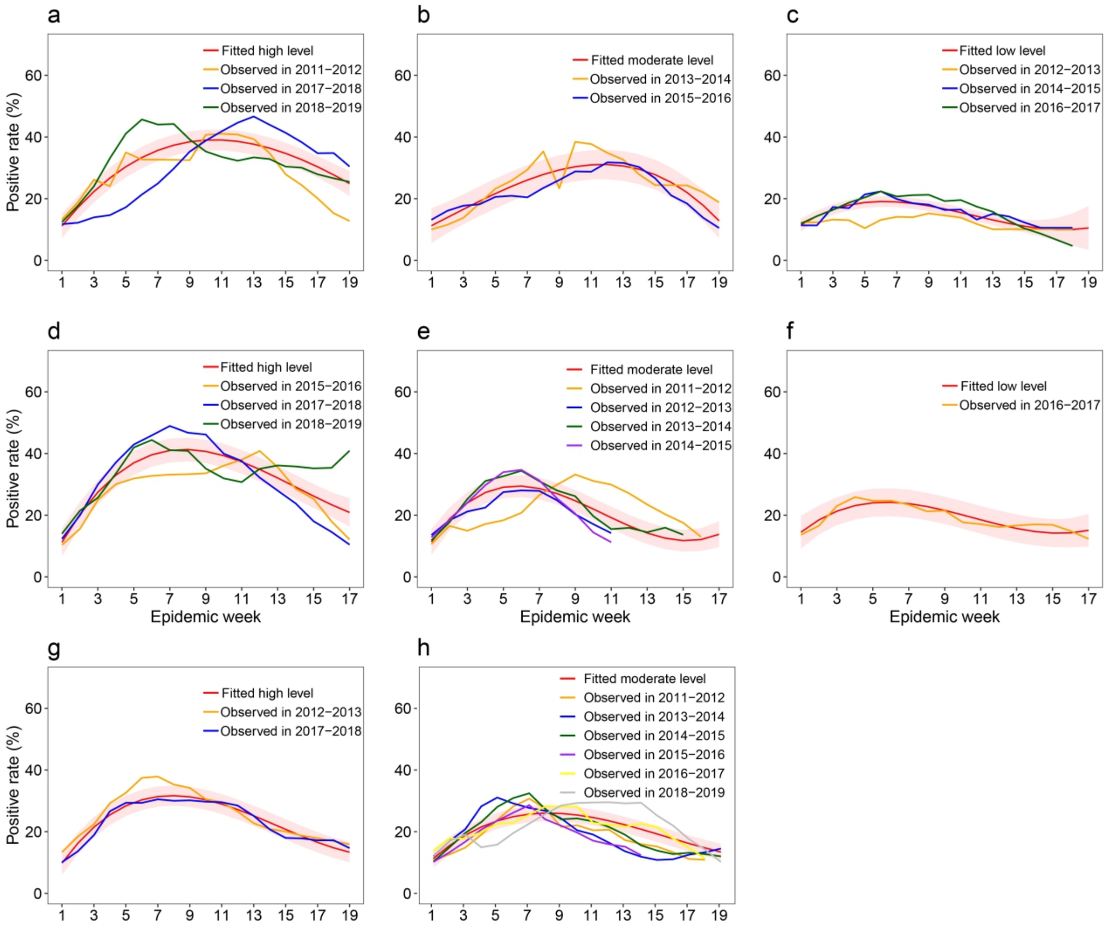

Supplementary Fig. 1. Influenza epidemic levels by region across 2011-2019. The fitted curve (red line) for each intensity level by region is presented with the lower and upper bounds (shaded pink color). The colored lines represent the observations of influenza activities in different years. **a** High epidemic intensity level in Southern China. **b** Moderate epidemic intensity level in Southern China. **c** Low epidemic intensity level in Southern China. **d** High epidemic intensity level in Northern China. **e** Moderate epidemic intensity level in Northern China. **f** Low epidemic intensity level in Northern China. **g** High epidemic intensity level in the US. **h** Moderate epidemic intensity level in the US.

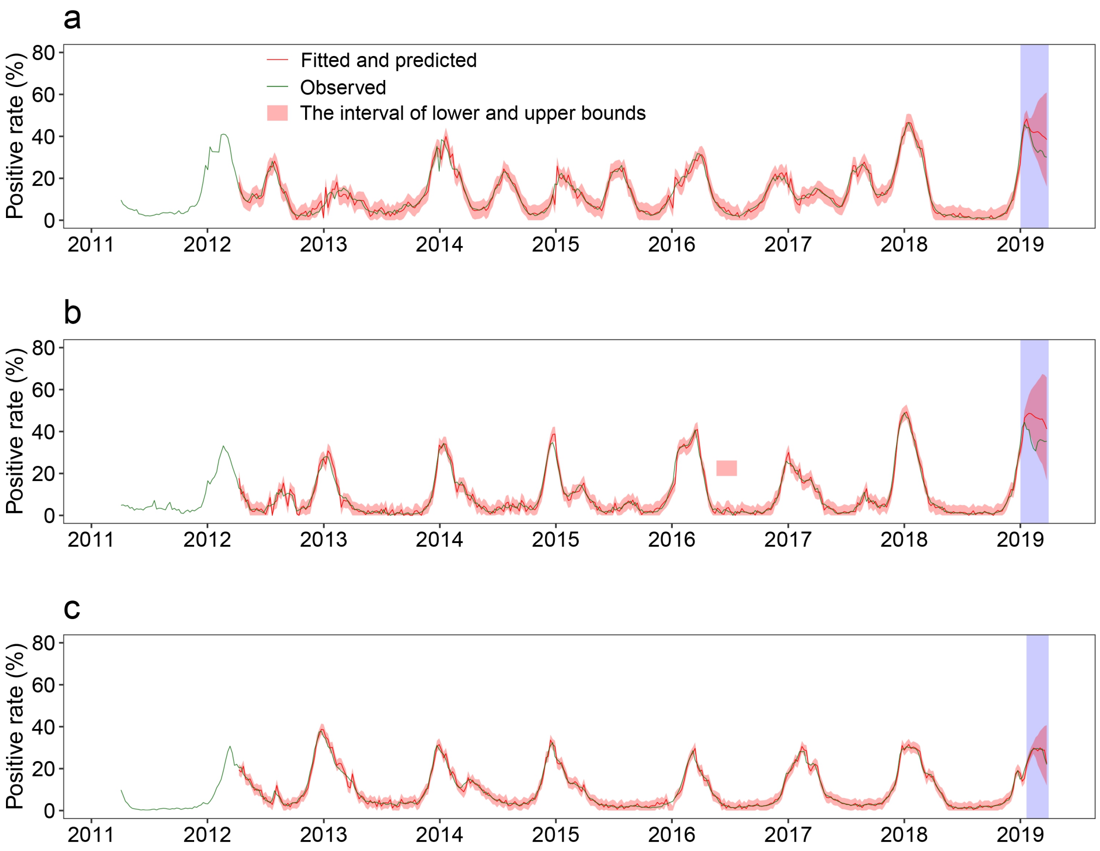

Supplementary Fig. 2. Actual and predicted positive rate from 2011 to 2019. The red lines represent the three processes of the ARIMA model's prediction degree. The blue shaded areas indicate actual and predicted positive rates in 2019 to identify the reliability and predictivity of the model. **a** Southern China. **b** Northern China. **c** the US.

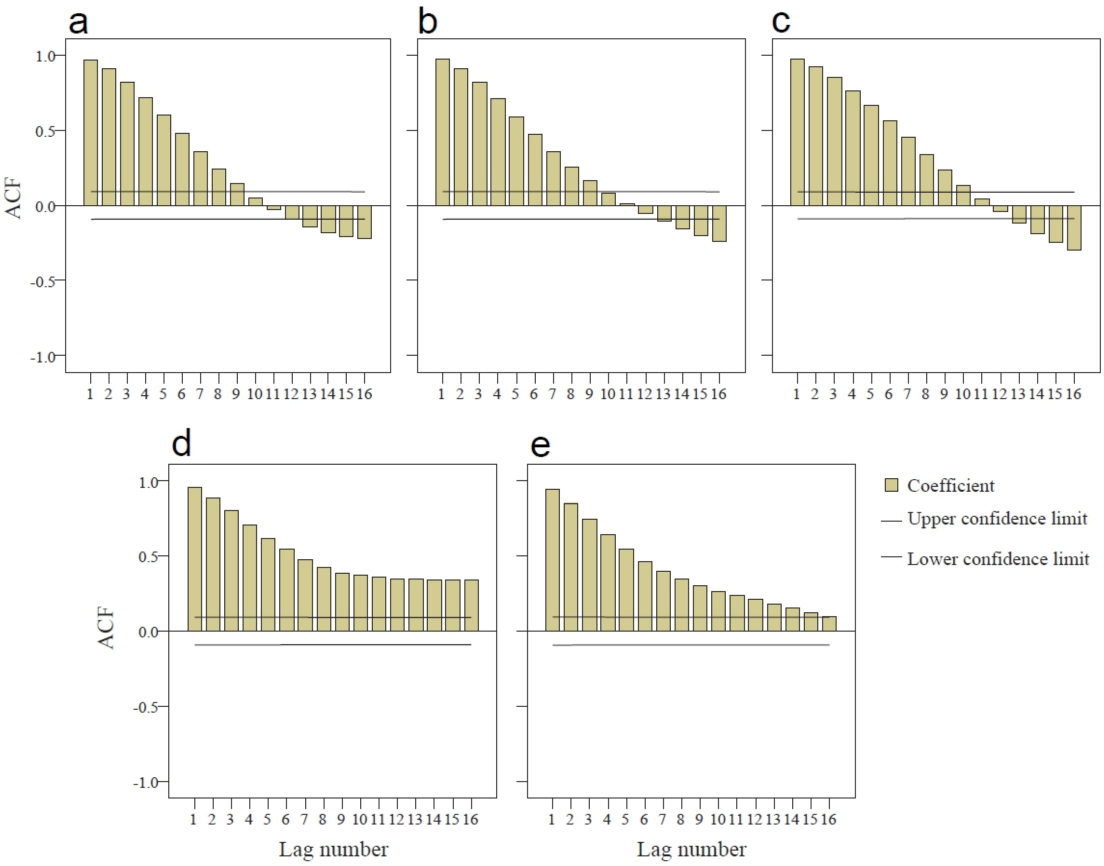

Supplementary Fig. 3. Stationarity test.

ACF plots are used to identify non-stationary time series. Only non-stationary series can be used for ARIMA models. Supplementary Fig. 3 shows that the ACF of non-stationary data decreases slowly while the ACF of a stationary time series will drop to zero relatively quickly, which supports that the time series is non-stationary. **a** Positive rate of influenza tests in Southern China. **b** Positive rate of influenza tests in Northern China. **c** Positive rate of influenza tests in the US. **d** Number of influenza-like cases in Southern China. **e** Number of influenza-like cases in Northern China.

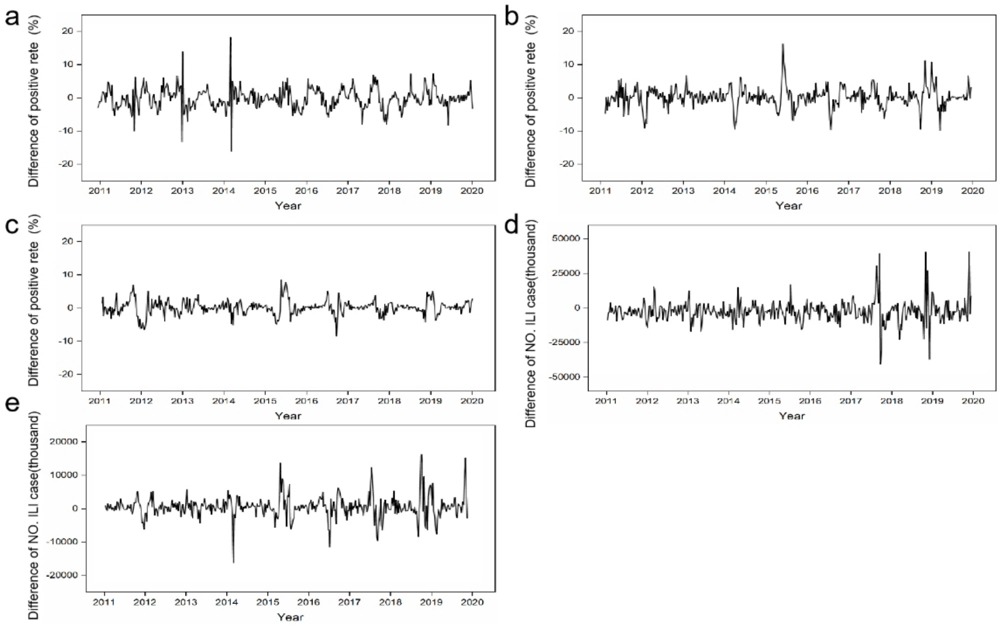

Supplementary Fig. 4. Difference.

As the sequence is non-stationary series, an initial differencing step can be applied to eliminate the non-stationarity of the mean function. The "difference" order is the integration order. It represents the number of times need to integrate the time series to ensure stationarity. After 1-time general difference and 1-time seasonal difference, the mean value fluctuates around zero as white noise. **a** Positive rate of influenza tests in Southern China. **b** Positive rate of influenza tests in Northern China. **c** Positive rate of influenza tests in the US. **d** Number of influenza-like cases in Southern China. **e** Number of influenza-like cases in Northern China.

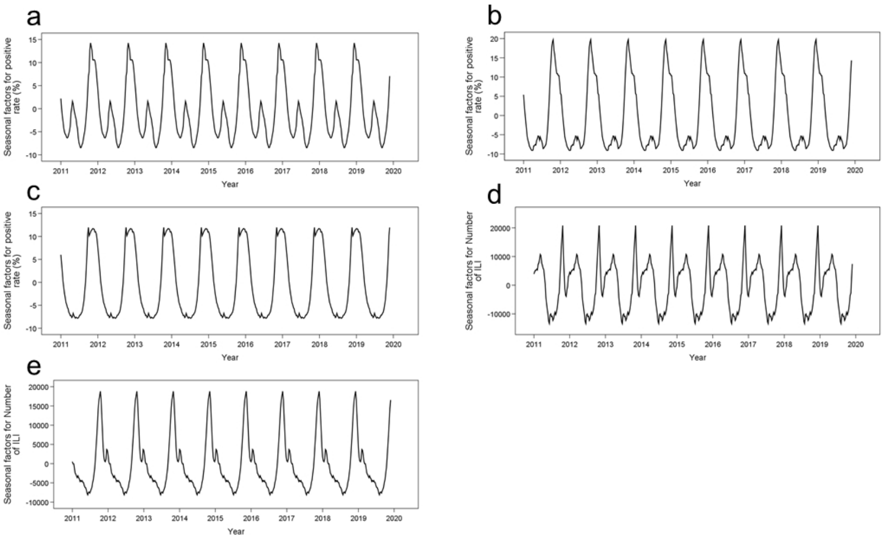

Supplementary Fig. 5. Seasonal adjustment factors (SAF).

The SAF refers to the seasonal factors decomposed from the series, in which the variable values are likely repeated according to the seasonal cycle. **a** Positive rate of influenza tests in Southern China. **b** Positive rate of influenza tests in Northern China. **c** Positive rate of influenza tests in the US. **d** Number of influenza-like cases in Southern China. **e** Number of influenza-like cases in Northern China.

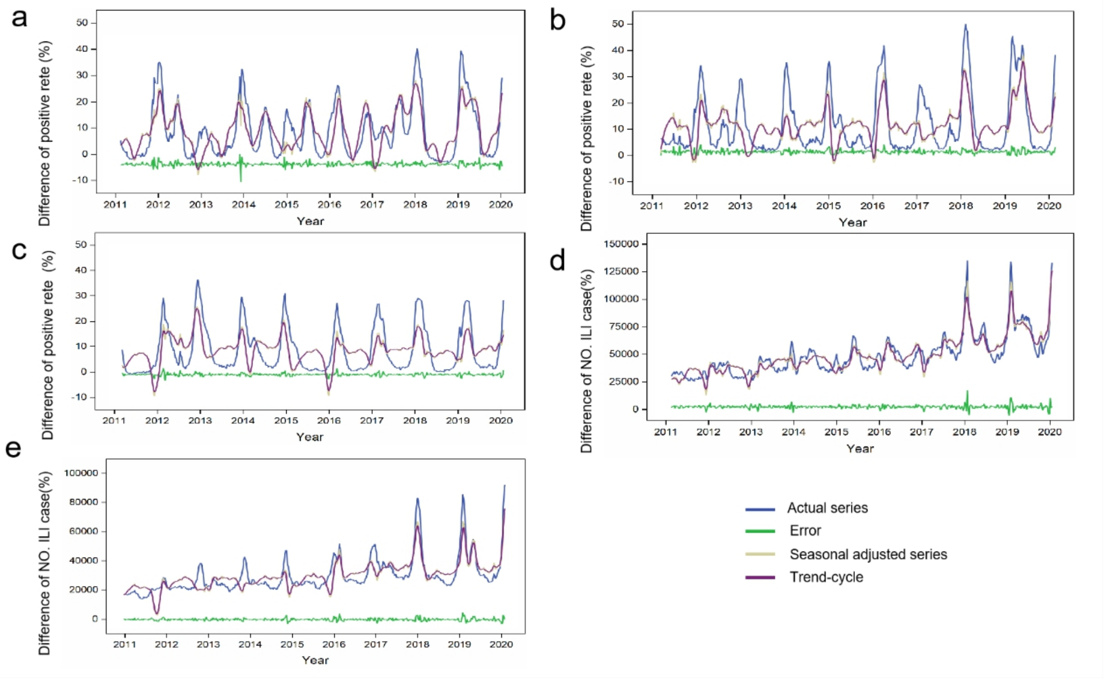

Supplementary Fig. 6. Seasonal Decomposition.

The ERR (error sequence) refers to the sequence left after removing seasonal factors, long-term trends, and cyclic changes from the time series - that is, the sequence composed of irregular changes in the original sequence. SAS (Seasonal adjusted series) refers to the correction sequence after removing the seasonal factors in the original sequence. STC (Trend-cycle) refers to the sequence consisted of long-term trends, and cyclic changes. **a** Positive rate of influenza tests in Southern China. **b** Positive rate of influenza tests in Northern China. **c** Positive rate of influenza tests in the US. **d** Number of influenza-like cases in Southern China. **e** Number of influenza-like cases in Northern China.

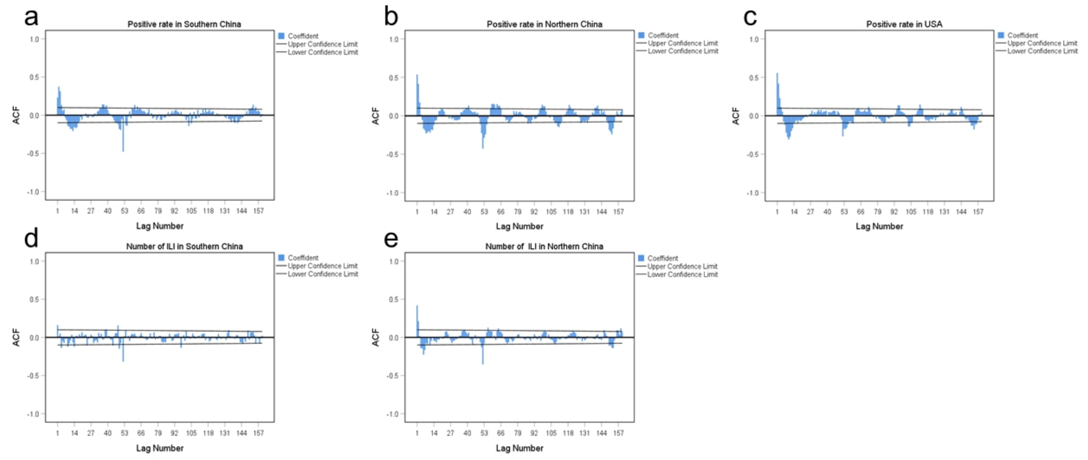

Supplementary Fig. 7. Auto-correlation function (ACF).

The ACF plot is a bar chart of the coefficients of correlation between a time series and self-lag. **a** Positive rate of influenza tests in Southern China. **b** Positive rate of influenza tests in Northern China. **c** Positive rate of influenza tests in the us. **d** Number of influenza-like cases in Southern China. **e** Number of influenza-like cases in Northern China.

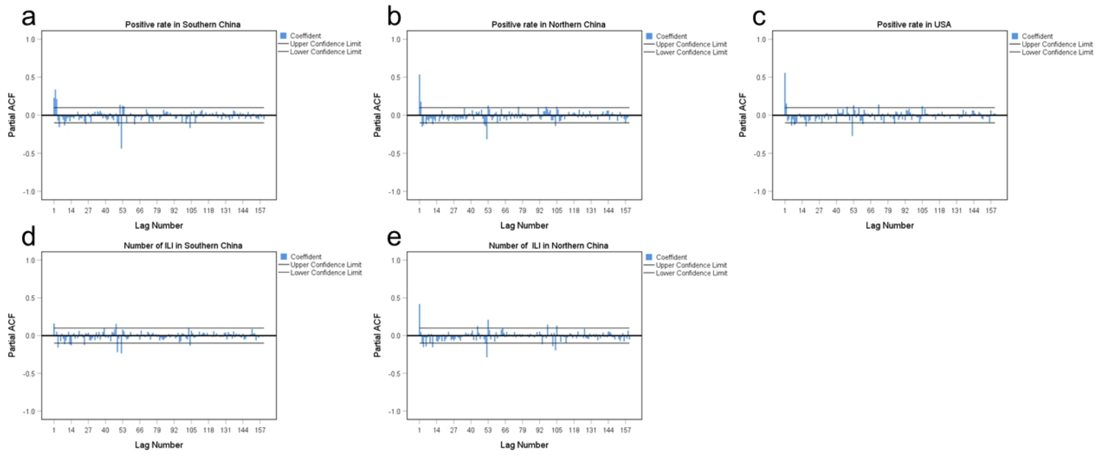

Supplementary Fig. 8. Partial auto-correlation function (PACF).

The PACF plot is a plot of the partial correlation coefficients between the series and self-lag. **a** Positive rate of influenza tests in Southern China. **b** Positive rate of influenza tests in Northern China. **c** Positive rate of influenza tests in the US. **d** Number of influenza-like cases in Southern China. **e** Number of influenza-like cases in Northern China.

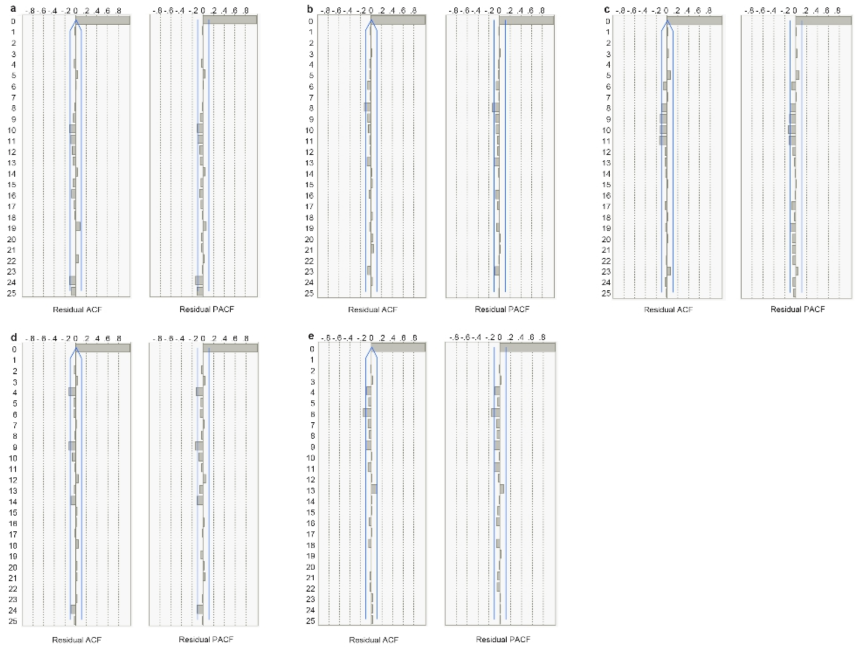

Supplementary Fig. 9. Partial auto-correlation function.

The ACF and PACF of residuals locate in the confidence interval and the mean value fluctuates around 0, which means that the ARIMA model is reasonable. If there is a correlation between residual of ACF and PACF, then there is still information left that can be used in the prediction. **a** Positive rate of influenza tests in Southern China. **b** Positive rate of influenza tests in Northern China. **c** Positive rate of influenza tests in the US. **d** Number of influenza-like cases in Southern China. **e** Number of influenza-like cases in Northern China.

Supplementary Tables

Supplementary Table 1. Polynomial fitting formula and goodness-of-fit for influenza activities.

Activity level	Southern China	R^2	Northern China	R^2	the US	R^2
High	$y=4.36+7.24x-0.44x^2-0.01x^3$	0.971	$y=0.19+12.08x-1.07x^2+0.03x^3$	0.972	$y=1.92+8.6x+-0.75x^2+0.02x^3$	0.969
Moderate	$y=8.64+2.56x-0.06x^2-0.01x^3$	0.923	$y=1.56+11.07x-1.32x^2+0.04x^3$	0.961	$y=6.59+5.05x+-0.38x^2+0.00715x^3$	0.961
Low	$y=7.80+4.14x-0.45x^2+0.01x^3$	0.952	$y=9.22+5.96x-0.71x^2+0.02x^3$	0.831	-	-

Supplementary Table 2. Parameterization and comparisons of ARIMA models.

Indicators	Forecasting influenza test positive rate			Forecasting ILI cases	
	Southern China	Northern China	The US	Southern China	Northern China
Parameters#	(1, 1, 3) (1, 1, 1)	(1, 1, 2) (0, 1, 2)	(1, 1, 2) (0, 1, 2)	(1, 1, 0) (0, 1, 1)	(1, 1, 2) (0, 1, 2)
Parameters 1	(1, 1, 2) (1, 1, 1)	(1, 1, 2) (1, 1, 1)	(1, 1, 2) (1, 1, 1)	(1, 1, 1) (0, 1, 1)	(1, 1, 2) (1, 1, 1)
Parameters 2	(1, 1, 3) (0, 1, 2)	(1, 1, 2) (0, 1, 1)	(1, 1, 2) (0, 1, 1)	(1, 1, 0) (0, 1, 2)	(1, 1, 2) (1, 1, 2)
AIC	1811.6	1719.9	1422.9	8027.8	7407.1
AIC1	1811.8	1720.3	1423.4	8029.7	7407.3
AIC2	1812.4	1720.6	1424.1	8029.8	7408.0
R^2	0.951	0.969	0.976	0.945	0.969
$R^2$1	0.951	0.969	0.976	0.945	0.969
$R^2$2	0.951	0.969	0.976	0.945	0.969

ILI: influenza-like illness.

#The optimized model parameters are listed in the table, and the top three models with best performance selected from multiple candidate models (https://zenodo.org/record/4573183#.YD5JWGgzZdg) are presented for comparisons. In this study, the optimal model was selected according to the principle of minimum AIC and maximum R^2.

Supplementary References

1. Liu, L. et al. Predicting the incidence of hand, foot and mouth disease in Sichuan province, China using the ARIMA model. *Epidemiol Infect* **144**, 144-151 (2016).
2. U.S. Centers for Disease Control and Prevention. Past Weekly Surveillance Reports. https://www.cdc.gov/flu/weekly/pastreports.htm. Accessed 30 Oct 2020.
3. Basakin, E. E. & Ekmekcioglu, O. Comment on "Comparison of the Ability of ARIMA, WNN and SVM Models for Drought Forecasting in the Sanjiang Plain, China". *Nat Resource Res* **29**,1465-1467 (2020).
4. Chowell,G., Tariq, A. & Hyman JM. A novel sub-epidemic modeling framework for short-term forecasting epidemic waves. *BMC Med* **17**, 164 (2019).
5. Lopes, R. F. L., Fraiha, S. G. C., Lima, V. D.,Gomes, H. S.&Cavalcante, G. P. S. Hybrid ARIMA and Neural Network Modelling Applied to Telecommunications in Urban Environments in the Amazon Region. International *Journal of Antennas and Propagation* **2020**,1-14 (2020).
6. Singh. R. K., et al. Prediction of the COVID-19 Pandemic for the Top 15 Affected Countries: Advanced Autoregressive Integrated Moving Average (ARIMA) Model. *JMIR Public Health Surveill* **6**, e19115 (2020).
7. Kane, M. J., Price, N., Scotch, M., & Rabinowitz, P. M. Comparison of ARIMA and Random Forest time series models for prediction of avian influenza H5N1 outbreaks. *BMC Bioinformatics* **15**, 276 (2014).
8. Wang, Y. & Guo, Y. K. Forecasting Method of Stock Market Volatility in Time Series Data Based on Mixed Model of ARIMA and XGBoost. *China Communications* **17**, 205-221 (2020).
9. Rehan, J. Hydroelectricity consumption forecast for Pakistan using ARIMA modeling and supply-demand analysis for the year 2030. *Renewable Energy* **154**, 46-51 (2020).

10. Zheng, A. et al. An application of ARIMA model for predicting total health expenditure in China from 1978-2022.*Journal of global health* **10**, 243-246 (2020).

11. Benvenuto, D., Giovanetti, M., Vassallo, L., Angeletti, S. &Ciccozzi, M. Application of the ARIMA model on the COVID-2019 epidemic dataset. *Data in brief* **29** (2020).
