# Supplementary material for: Impact of COVID-19 outbreaks and interventions on influenza in China and the United States
Source: Nat Commun. 2021 May 31;12:3249. doi: 10.1038/s41467-021-23440-1 (PMC8167168; doi:10.1038/s41467-021-23440-1)
Supplement: Supplementary file 2 — Reporting Summary [file 41467_2021_23440_MOESM2_ESM.pdf]

nature research

Corresponding author(s): Zhongjie Li, George F. Gao

Last updated by author(s): Apr 21, 2021

Reporting Summary

Nature Research wishes to improve the reproducibility of the work that we publish. This form provides structure for consistency and transparency in reporting. For further information on Nature Research policies, see our Editorial Policies and the Editorial Policy Checklist.

Statistics

For all statistical analyses, confirm that the following items are present in the figure legend, table legend, main text, or Methods section.

n/a	Confirmed	
☒	☐	The exact sample size (*n*) for each experimental group/condition, given as a discrete number and unit of measurement
☐	☒	A statement on whether measurements were taken from distinct samples or whether the same sample was measured repeatedly
☐	☒	The statistical test(s) used AND whether they are one- or two-sided<br>*Only common tests should be described solely by name; describe more complex techniques in the Methods section.*
☒	☐	A description of all covariates tested
☐	☒	A description of any assumptions or corrections, such as tests of normality and adjustment for multiple comparisons
☐	☒	A full description of the statistical parameters including central tendency (e.g. means) or other basic estimates (e.g. regression coefficient) AND variation (e.g. standard deviation) or associated estimates of uncertainty (e.g. confidence intervals)
☐	☒	For null hypothesis testing, the test statistic (e.g. *F*, *t*, *r*) with confidence intervals, effect sizes, degrees of freedom and *P* value noted<br>*Give P values as exact values whenever suitable.*
☒	☐	For Bayesian analysis, information on the choice of priors and Markov chain Monte Carlo settings
☒	☐	For hierarchical and complex designs, identification of the appropriate level for tests and full reporting of outcomes
☒	☐	Estimates of effect sizes (e.g. Cohen's *d*, Pearson's *r*), indicating how they were calculated

Our web collection on statistics for biologists contains articles on many of the points above.

Software and code

Policy information about availability of computer code

Data collection	We downloaded virological and ILI surveillance online data in China from the National Influenza Surveillance Network in 2011–2020(http://www.chinaivdc.cn/cnic/). The National Influenza Surveillance Network in mainland China, led by China CDC, has 554 sentinel hospitals and 407 network laboratories. Influenza activity levels and trends are monitored using ILI data from surveillance units collected at sentinel hospitals. The Influenza Network Laboratory monitors the etiology of influenza virus from respiratory specimens, which not only include ILI patients from influenza surveillance sentinel hospitals but also include samples collected during influenza outbreaks. In China, weekly virological and ILI data, based on influenza sentinel surveillance, are systematically collected as a proxy of influenza activity. Every 12 month interval, from the 14th week in one year to the 13th week of the following year constitute a surveillance year.<br>We downloaded publicly available influenza virological data in 2011–2020 released by US CDC as well (https://www.cdc.gov/flu/weekly/index.htm). In the US, the Influenza Surveillance Network, led by US CDC, contains about 100 public health laboratories and over 300 clinical laboratories. Clinical laboratories primarily test respiratory specimens for diagnostic purposes and provide information on the timing and intensity of influenza activity. Public health laboratories test specimens from clinical laboratories for surveillance purposes to understand influenza virological information such as the virus types, subtypes, and lineages that are circulating. The total number of respiratory specimens tested for influenza and the number positive for influenza viruses are reported from public health and clinical laboratories to CDC each week.<br>The positive test rate of influenza in China was calculated from a total of 3,728,252 samples; the positive test rate for the US was determined from a total of 8,349,337 samples over 9 years.
Data analysis	We compared fitted activity levels in 2011-2019 with observed activity in the winter-spring epidemic weeks in 2019-2020 before the COVID-19 outbreaks and the implementation of NPIs. We then determined predicted influenza activity by intensity level under a counterfactual scenario of no COVID-19. We investigated influenza infections based on key dates for NPIs in China and the US: January 23, 2020 – Wuhan's lockdown – as the start of strict and combined NPIs in China; March 13, 2020 – when a state of national emergency was declared by the US–as the start of NPIs in the US.

Time series models. The ARIMA (p, d, q) model is a time series forecasting method developed by British-born American statistician George E. P. Box and the British statistician Gwilym M.Jenkins in the early 1970s. ARIMA is an extension of autoregressive (AR), moving average (MA), and ARMA models20 36. It aims to solve two problems: one is to decompose randomness, stationarity, and seasonality of time series; the other is to select an appropriate model for forecasting based on analysis of time series. ARIMA has been widely used to forecast short-term effects and trends of acute infectious diseases36. The parameters p, d, and q represent the order of autoregressive (AR), the degree of differencing of the original time series, and the order of the moving average (MA), respectively. Due to the seasonality of influenza, we utilized a seasonal ARIMA (SARIMA [p, d, q][P, D, Q]s) model. In SARIMA, P, D, Q, and s refer to seasonal autoregression, seasonal integration, seasonal moving average, and seasonal period length.

a) Sequence stationarity. Time sequences (test positivity rates in Southern and Northern China and the U.S., and the number of ILI cases in Southern and Northern China) were nonstationary (Supplementary Information Figure S3). Sequence stationarity was tested with the augmented Dickey–Fuller (ADF) test. If lags were outside the confidence intervals after the first three lags, the time sequence was considered nonstationary. After 1-time difference and 1-time seasonal difference, the data sequence is stable with the mean value fluctuating around the indication. (Supplementary Information Figure S4).

b) Sequence randomness. According to the Box-Ljung statistical test results (p<0.05), the hypotheses of independence of the 5-time sequences were all rejected.

c) Identification. Depending on the seasonal decomposition, SAF (seasonal adjustment factors), referring to factors of the seasonal cycle that affect the sequence (Supplementary Information Figure S5). ERR (error sequence), referring to the sequence remaining after removing seasonal factors, long-term trends, and cyclic changes from the time series, was around zero (within 5) and distributed as white noise (Supplementary Information Figure S6).

Through observing the autocorrelation function (ACF) (Supplementary Information Figure S7) and partial autocorrelation function (PACF) (Supplementary Information Figure S8) to recognize and analyze the characteristics of the sequence, we first listed the parameters that met the characteristic of ACF and PACF, and then optimized the parameters in accordance with Akaike information criterion (AIC) and R2. Additionally, autoregressive model (AR) describes the relationship between the current value and the historical value. Since the positive rate of influenza is related to the characteristics of the virus in the epidemic season and the serial interval of influenza is 2-3 days7, AR was selected as order 1. Generally, as the duration of influenza immunity antibody is less than one year37, it may affect the intensity of influenza activity in the next year. We chose 0-1 for seasonal autocorrelation, but we only presented the top three candidate models in the Supplementary Information Table S2.

d) Estimation and validation. Rationality of the model was assessed by examination of standard model fitting residuals. If fitting residuals of a model for sequences of this study were normally distributed with zero as the mean, and the lag order residuals of ACF and PACF were within confidence intervals (Supplementary Information Figure S9), the model was regarded as qualified. To further validate the predictive ability of the model, we also used the influenza data from 2011 to 2018 as a training set to build models and predict the influenza activities for the 2018-2019 season. Results were assessed by comparing the test dataset of observed values in 2018-2019 and the mean absolute percentage errors. (Supplementary Information Methods and Figure S2).

e) Application forecasting. We used these models with data from 2011-2019 to estimate the weekly influenza positivity rate for the winter-spring season in 2019-2020 under a counterfactual scenario with no COVID-19 outbreaks and no COVID-19 NPIs. For China, forecasting started from the week of January 7, 2020 when the SARS-CoV-2 was first identified, corresponding to epidemic week 8 in Southern China and epidemic week 6 in Northern China. For the US, the first week for estimating was the week beginning on January 20, 2020, corresponding to epidemic week 10 in the US. The overall impact of COVID-19 outbreaks and interventions on influenza was defined as the difference in the area between the observed epidemic curve and the model-predicted curve. The upper/lower bounds of estimates were defined as the difference between the observed curve and the model-predicted upper/lower bound curve of confidence intervals. We also assessed the effectiveness of COVID-19 outbreaks and interventions by time period (Table 1), according to the timings of first identification of SARS-CoV-2 and the implementation of strict NPIs in China, and the dates of the first COVID-19 confirmed case reported and the national emergency declared in the US. Descriptive statistics and time series analyses were conducted using SAS JMP Pro 14 and SPSS 22.0. The 2019-2020 curve area difference for assessing the NPIs effectiveness used Graphpad prism 8.0. R version 3.6.1 (R Foundation and Origin 2019 for Statistical Computing, Vienna, Austria) was used to plot figures.

For manuscripts utilizing custom algorithms or software that are central to the research but not yet described in published literature, software must be made available to editors and reviewers. We strongly encourage code deposition in a community repository (e.g. GitHub). See the Nature Research guidelines for submitting code & software for further information.

Data

Policy information about availability of data

All manuscripts must include a data availability statement. This statement should provide the following information, where applicable:

- Accession codes, unique identifiers, or web links for publicly available datasets
- A list of figures that have associated raw data
- A description of any restrictions on data availability

The influenza virological surveillance data in the US used in this study are publicly available at: https://www.cdc.gov/flu/weekly/fluactivitysurv.htm. All other data associated with this work are available at https://zenodo.org/record/4573183#.YD5JWGgzZdg

Field-specific reporting

Please select the one below that is the best fit for your research. If you are not sure, read the appropriate sections before making your selection.

☒ Life sciences ☐ Behavioural & social sciences ☐ Ecological, evolutionary & environmental sciences

For a reference copy of the document with all sections, see nature.com/documents/nr-reporting-summary-flat.pdf

Life sciences study design

All studies must disclose on these points even when the disclosure is negative.

Sample size	This study did not involve sampling, it analyzed influenza surveillance data in China and the US during the 2011-2020 surveillance year.<br>The National Influenza Surveillance Network in mainland China, led by China CDC, has 554 sentinel hospitals and 407 network laboratories. Influenza activity levels and trends are monitored using ILI data from surveillance units collected at sentinel hospitals. The Influenza Network Laboratory monitors the etiology of influenza virus from respiratory specimens, which not only include ILI patients from influenza surveillance sentinel hospitals but also include samples collected during influenza outbreaks.<br>In the US, the Influenza Surveillance Network, led by US CDC, contains about 100 public health laboratories and over 300 clinical laboratories13. Clinical laboratories primarily test respiratory specimens for diagnostic purposes and provide information on the timing and intensity of influenza activity. Public health laboratories test specimens from clinical laboratories for surveillance purposes to understand influenza virological information such as the virus types, subtypes, and lineages that are circulating. The total number of respiratory specimens tested for influenza and the number positive for influenza viruses are reported from public health and clinical laboratories to CDC each week.<br>Data collection is representative in terms of the scope, quantity and distribution of surveillance areas.
Data exclusions	We excluded incomplete samples of influenza tested in advance to ensure that the data will not be duplicated
Replication	To further validate the predictive ability of the model, we used the influenza data from 2011 to 2018 as a training set to build models and predict the influenza activities for 2018-2019 season. Then they were assessed by using the test dataset of observed values in 2018-2019 and the average absolute error. We selected the most optimal model with the lowest value in Akaike information criterion (AIC) and the coefficient of determination that shows the goodness of model fit, from the candidate models. Descriptive statistics and time series analyses were conducted using SAS JMP Pro 14 and SPSS 22.0. The 2019-2020 curve area difference for assessing the NPIs effectiveness used Graphpad prism 8.0. R version 3.6.1 (R Foundation and Origin 2019 for Statistical Computing, Vienna, Austria) was used to plot figures. The influenza virological surveillance data in the US used in this study are publicly available at: https://www.cdc.gov/flu/weekly/fluactivitysurv.htm. All other data associated with this work are available at https://zenodo.org/record/4573183#.YD5JWGgzZdg
Randomization	Sentinel hospitals cover all cities in China rather than concentrated in economically developed areas, so the sources of data are random. In addition, the method of surveillance is in a passive manner.
Blinding	The acquisition of data depended on passive reporting by the surveillance system. Researchers could not distinguish the NPIs information of reported cases, and there is no selection bias, so it is blind.

Reporting for specific materials, systems and methods

We require information from authors about some types of materials, experimental systems and methods used in many studies. Here, indicate whether each material, system or method listed is relevant to your study. If you are not sure if a list item applies to your research, read the appropriate section before selecting a response.

Materials & experimental systems

n/a	Involved in the study	
☒	☐	Antibodies
☒	☐	Eukaryotic cell lines
☒	☐	Palaeontology and archaeology
☒	☐	Animals and other organisms
☒	☐	Human research participants
☒	☐	Clinical data
☒	☐	Dual use research of concern

Methods

n/a	Involved in the study	
☒	☐	ChIP-seq
☒	☐	Flow cytometry
☒	☐	MRI-based neuroimaging
